# Ranney on Nervous Diseases

**Published:** 1889-07

**Authors:** Ambrose L. Ranney

**Affiliations:** 156 Madison Avenue, New York City


### *RANNEY ON NERVOUS DISEASES.*

*To the Editor of the Buffalo Medical and Surgical Journal:*

SIR—My attention has been drawn to a very courteous and complimentary review of my recent work on Nervous Diseases, in the June number of your journal.

I find in it, however, a statement that is liable to mislead your readers and which certainly misstates my views. I refer to the following sentence:

"Especially does he (*i. e.*, myself,) pin his faith to the influence of *refractive errors* in chorea and epilepsy." [1]

I take the liberty, in answer to this unintentional error on the part of the reviewer, of quoting some conclusions that I embodied in a late article on "Eye-strain." These conclusions are as follows:

1st. "Eye-strain" arises chiefly from defects in the *refraction of the eye and an imperfect equilibrium in the muscles which move the eyes.*

2d. These conditions, when present, tend to cause an excessive expenditure of nerve-force by the individual in direct proportion to the amount of defect to be overcome.

3d. Excessive expenditure of nerve-force upon any one organ is commonly made at the expense of some other organ; or, if not, is paid out of the "reserve" of nerve capital possessed by the individual.

4th. The extent of the drafts thus made upon the "reserve" capital and the amount of "reserve capital" are the two factors which can alone determine, in any individual case, how long this state of things can last without causing a "nervous bankruptcy."

5th. The conditions mentioned as those which chiefly tend to

1. Italics are my own.

cause " eye-strain " are transmitted from parent to child; hence they become operative at birth, and last until death, unless mechanically or otherwise relieved.

6th. They are capable of detection and accurate measurement during life by scientific procedures. Some of the methods employed by oculists in *testing the eye muscles* are not worthy of perpetuation.

7th. A condition of exhausted nervous vitality is sure to impair the general health in many ways, and to render the individual man more liable to disease than when in full vigor.

8th. Many of the constitutional diseases which ultimately imperil the lives of their victims, are indirectly the result of a *state of low nervous vitality* (a state which is frequently the result of " eye-strain," from well-understood causes that might have been easily recognized and relieved).

9th. The so-called " inherited predisposition " to certain diseases is unquestionably based, in many cases, upon some anomaly of the visual apparatus. I am so well convinced of this fact that I assert it without fear of contradiction, from carefully gathered statistics.

10th. The examination of the eye for errors of refraction and accommodation, and a thorough familiarity with the tests lately advocated for the detection of anomalies of the ocular muscles, ought not to be confined exclusively to the practice of the oculist.

They are as valuable to the general practitioner as are the physical signs of the chest.

By giving these facts due prominence in your next issue, you will greatly oblige, Yours most respectfully,

AMBROSE L. RANNEY, M. D.

156 MADISON AVENUE, NEW YORK CITY, June 12, 1889.

---

PREFERRED CREDITORS.—Medical men in general are probably not aware that in France the doctors' claim on the estate of a deceased patient has precedence of all others. Even the landlords' claims for arrears of rent must yield to the doctors' fee. The courts have decided that as it is an imperative right of humanity that the dying should have the necessary care and treatment, such attendance should be paid for before all the other debts. Such a law in this country would be hailed with satisfaction by the doctors, and a similar provision for the undertakers would delight that profession.—*Scientific American*, May 4th.

